# Five Ways to Cure a Cold

**Published:** 1891-02

**Authors:** 


Five Ways to Cure a Cold.—1. Bathe the feet in hot water and take a pint of hot lemonade. Then sponge with salt water and remain in a warm room. 2. Bathe the face in very hot water every five minutes for an hour. 3. Snuff up the nostrils hot salt water every three hours. 4. Inhale ammonia or menthol. 5. Take four hours active exercise in the air. A ten grain dose of quinine will usually break up a cold in the beginning. Any thing that will set the blood in active circulation will do it, whether it be drugs, or the use of a bucksaw.

